# Challenges in the management of a patient with Cowden syndrome: case report and literature review

**DOI:** 10.1186/1897-4287-10-5

**Published:** 2012-04-14

**Authors:** Inga Melbārde-Gorkuša, Arvīds Irmejs, Dace Bērziņa, Ilze Štrumfa, Arnis Āboliņš, Andris Gardovskis, Signe Subatniece, Genādijs Trofimovičs, Jānis Gardovskis, Edvīns Miklaševičs

**Affiliations:** 1 Hereditary Cancer Institute, Rīga Stradiņš University, Dzirciema Street 16, LV-1007 Riga, Latvia; 2 Breast Unit, Pauls Stradins Clinical University Hospital, Pilsonu Street 13, LV-1002 Riga, Latvia

**Keywords:** Cowden syndrome, PTEN, Cancer, Risk-reducing mastectomy

## Abstract

We would like to present a patient with a classical phenotype of a rare disorder - Cowden syndrome, its diagnostics and management challenges. A breast surgeon has to be aware of this rare condition when treating a patient with breast manifestations of Cowden syndrome and has to refer the patient to a clinical geneticist for further evaluation. Sequencing of the *PTEN *gene showed the Asp24Gly mutation. According to the latest literature data, the lifetime risk of breast cancer for Cowden syndrome patients is 81% and surgery is a justified option to reduce the risk of breast cancer. Bilateral risk-reducing mastectomy with immediate reconstruction was performed to eliminate further risk of breast cancer. 3 years after the risk-reducing breast surgery the patient is satisfied with the outcome. This is to our best knowledge the first reported Cowden syndrome case with follow-up data after risk-reducing measures have been taken.

## Background

Cowden syndrome (CS) is an uncommon disorder with an estimated incidence of 1 per 200,000 of the population, characterized by germline mutations in the tumor suppressor gene *PTEN *[1,2]. CS is associated with highly variable symptoms and signs, meaning that it might be underdiagnosed; in addition, approximately 20% of CS patients have no identified genetic explanation of their phenotypic features [3].

CS is characterized by multiple hamartomas in a variety of tissues and indicates an increased risk of developing malignancies. In female patients the syndrome is associated with up to 50% lifetime risk of developing breast cancer, 5-10% risk of developing endometrial cancer, and 10% lifetime risk of developing follicular thyroid cancer [4]. In recent reports the elevated lifetime cancer risk in Cowden syndrome patients or in individuals with germline *PTEN *mutations is up to 85% for breast cancer, up to 35% for thyroid cancer, and up to 28% for endometrial cancer [5,6]. Lifetime cancer risks are now extending to kidney cancer and colorectal cancer (up to 33% and 16%, respectively).

Other features of the disorder include macrocephaly, gastrointestinal polyps, benign breast, thyroid and endometrial manifestations, and characteristic mucocutaneous lesions. The diagnosis of CS in most cases is primarily based on the presence of mucocutaneous features, estimated to have 99% penetrance before the age of 30 [7]. A correlation between an identified *PTEN *mutation and breast cancer diagnosis in CS has been reported [3]. Breast cancers are most often diagnosed in CS patients at the age of 38-46 [8]. 34% of female CS patients diagnosed with breast cancer had bilateral diseases [5].

## Case presentation

The proband is a female born in Latvia in 1973. She has a remarkable medical history from the age of 32 when she underwent total thyroidectomy due to a benign nodular goiter. Cavernous angioma of the right temporal lobe (2.1 × 1.7 cm) was present on brain MRI with contrast and liver angioma was detected at a size of 0.8 cm on abdominal ultrasound at the age of 33.

The patient also suffered from hypochromic anemia secondary to metrorrhagia. Stage IA endometrial adenocarcinomawas detected at 34 and a following hysterectomy with bilateral salpingo-oophorectomy was performed.

Gastroscopy at the age of 34 revealed numerous asymptomatic polyps 6 cm below the cardia level (4-10 mm in size). Biopsies from the polyps were taken, histology showed hyperplastic polyps without malignancy.

**Figure 1:**
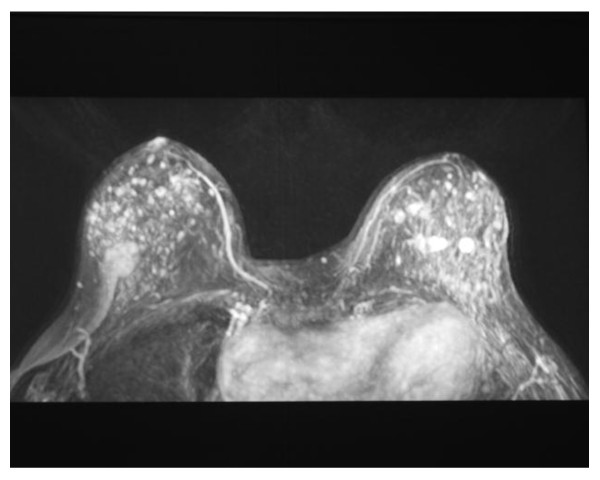
**T1-weighted breast MRI**. Representation of well-circumscribed, oval, multifocal, bilateral high signal intensity lesions.

At the age of 35 she visited a breast surgeon, complaining of palpable breast lesions. She had already had several bilateral breast fibroadenoma surgeries in the past. T1-weighed breast MRI (Figure 1) showed multiple, well-circumscribed, oval, bilateral high signal intensity lesions.

The clinical geneticist considered an initial diagnosis of CS according to the diagnostic criteria updated by the US NCCN 2008 [5]. The diagnosis was based on the presence of two major and three minor criteria (Table 1). The major criteria were endometrial carcinoma and macrocephaly. The exhibited minor criteria were nodular goiter, lipomas, and gastric polyps. Hamartomatous mucocutaneous lesions in the Latvian proband were not confirmed pathologically. A similar case has been described where the typical skin features were not reported before 55 years of age, whereas abnormal findings in several organs at a relatively young age were indicative [9]. Only focused examination revealed subtle skin lesions that are also widely spread in the general population. In our case, the presence of endometrial malignancy at such an early age was striking, and it was indicative of possible inheritance. Another significant factor was bilateral exuberant breast lesions.

**Table 1:** Presence of the diagnostic criteria of Cowden syndrome in proband according to US National Comprehensive Cancer Network (2008)

Pathognomonic criteria	Lhermitte-Duclos disease (dysplastic cerebellar gangliocytoma)	no
	Mucocutaneous lesions (trichilemmomas, acral keratoses, papillomatous lesions, mucosal lesions)	no
Major criteria	Breast cancer	no
	Thyroid cancer (especially follicular)	no
	Endometrial cancer	yes
	Macrocephaly (≥ 97% percentile)	yes
Minor criteria	Other thyroid lesions (e.g., adenoma, multinodular goiter)	yes
	Mental retardation (IQ ≤ 75)	no
	Gastrointestinal hamartomas	yes
	Fibrocystic breast disease	no
	Lipomas	yes
	Fibromas	no
	Genitourinary tumors (especially renal cell carcinoma)	no
	Genitourinary structural malformations	no
	Uterine fibroids	no

The diagnosis was further supported by the presence of the missense mutation Asp24Gly in the *PTEN *gene. DNA was isolated from blood using the FlexiGene (Qiagen) kit and the *PTEN *gene was sequenced as described earlier [10]. No clinically suspected or genetically established cases of CS among the 2,070,000 inhabitants of Latvia have been reported so far.

The family history of the proband did not reveal any significant pathology, a DNA sample was taken from the proband's parents and one of the two sibs who all appeared to be non-carriers of the mutation. Consequently, we can assume that the proband has received a *de novo *germline mutation, but it is impossible to establish its either paternal or maternal origin. The proband's level of intelligence is standard, she has socially active parents and two sisters.

Preoperative ultrasound-guided breast fine-needle aspiration from several abnormal areas was obtained, but no malignancy was detected.

Considering high future risk of breast cancer, prophylactic bilateral mastectomy was recommended by the multidisciplinary team as the most effective available risk-reducing measure for breast cancer. The indications of risk-reducing mastectomy and alternative management options were discussed with the patient. The patient chose to undergo a risk-reducing surgery. Bilateral skin-sparing mastectomy with immediate two stage expander/implant reconstruction was successfully performed. At the first stage, immediately after skin-sparing mastectomy, tissue expanders (Eurosilicone Vertex tissue expander, 500 cc) were placed in a complete submuscular position under the pectoralis major muscle. Partially for coverage the serratus anterior and pectoralis minor muscles were elevated. Intraoperatively, each tissue expander was filled with 200 ml saline.

The surgical specimen revealed mastopathy areas with features of fibroadenoma, areas of ductal hyperplasy, fibrosing adenosis, apocrine metaplasia, microcalcinates, and intracanalicular fibroadenoma.

During 6 weeks the expansion of tissue expanders was started biweekly with 50 - 100 ml until 550 ml were reached in each side.

Eight months later the expanders were replaced by permanent implants (Eurosilicone Cristalline Vertex Paragel, 102 N, high profile, 500 cc). Finally, nipple reconstruction using C-V flap technique was performed seven months after the placement of the implants. A three-year follow-up shows good clinical results and the proband feels well. Screening gastroscopy, colonoscopy, and abdominal CT scan were performed. Polyps were found only in the stomach and duodenum, histopathologic examination of biopsies showed hyperplastic polyps. Abdominal CT scan revealed that liver hemangioma had increased in size up to 3.5 cm and a new hemangioma of 3.3 cm in size was detected.

## Discussion

Because of the rarity of Cowden syndrome the management examples are insufficient in existing literature. Treatment for the benign and malignant manifestations in CS patients is the same as for the sporadic counterparts in the general population. The management of an individual with a *PTEN *mutation relates to a significant aspect - increased cancer surveillance, regarding the predisposition to a variety of cancers. The National Cancer Comprehensive Network (NCCN) issues are the most widely approved guidelines for the management of cancer risk in individuals with CS.

Recent studies have reported gastrointestinal polyps in 93% of *PTEN *mutation carriers and an increased lifetime risk for colorectal cancer of up to 16% in patients with CS [5,11]. Half of the CS patients have hyperplastic polyps [12], as in this case, suggesting that routine colonoscopy should be considered.

Elevated lifetime risk (15% and 33.6%) for renal cancer has also been found [5,6]. The authors recommended annual abdominal ultrasound or CT scanning and urinalysis.

Breast cancer is the most common component cancer of CS. Pilarski et al., reporting on the largest single cohort of *PTEN-*positive patients published to that date, found breast cancer in 32% (28/90) of female patients, with the mean age at molecular testing of 47.6 years (range 33-64 years) [13]. Recently, Tan et al. reports estimated the lifetime risk for breast cancer in patients with a *PTEN *mutation at 85.2% [6]. Similarly, Riegert-Johnson et al. ascertained breast cancer risk at 81% in patients meeting accepted diagnostic criteria for CS [5]. These two studies included a large cohort of patients (respectively, 368 and 210) for the first time and reported significantly higher cumulative lifetime risks than those previously published. Risk of breast cancer up to 80% is usually observed in *BRCA1 *and *BRCA2 *mutation carriers [14,15].

The presented patient is the only carrier of the Asp24Gly mutation in the *PTEN *gene among her tested family members (father, mother and sister) and the only one who is affected by CS. Obviously, this is not sufficient to certainly identify this mutation as a cause of CS but it should be noted that in tumors of various localizations (central nervous system, endometrium, intestine, ovary and urinary tract) somatic mutations in this position (D24G, D24E, D24Y and D24N) have been found (COSMIC). This mutation was predicted to be possibly damaging (score 0.539) by PolyPhen-2 analysis.

To control the possible development of breast cancer for CS patients without a family history, the NCCN recommends mammography and breast magnetic resonance imaging to be commenced at the age of 30-35 (or 5-10 years before the earliest known breast cancer in the family or whichever is earlier) [5]. The analysis of medical publications shows that 7% of CS breast cancers occurred prior to the recommended onset of radiographic screening by NCCN [5].

Breast cancer prevention also involves removal of the breast prior to the development of malignancy. At present, the most frequent indications for bilateral risk-reducing mastectomy are high risk histology (such as a typical ductal or lobular hyperplasia and lobular carcinoma *in situ*), strong family history and positive mutation in the *BRCA1 *or *BRCA2 *genes [16]. Although there are no data regarding risk-reducing surgery in females with Cowden syndrome, the option of risk-reducing mastectomy should be discussed on a case-to-case basis. It has been proven [17] that bilateral prophylactic mastectomy reduces the risk of breast cancer by 90% in women with intact ovaries and by 95% in women who have undergone both prophylactic mastectomy and oophorectomy in the case of *BRCA1/2 *gene mutation carriers. Only two cases of bilateral risk-reducing mastectomy for CS patients with no breast lesions where *PTEN *mutation is genetically confirmed have been described thus far [18].

The hamartomatous lesions of the breasts for CS patients are frequently diffuse, multifocal, bilateral and feature various complex histopathologic abnormalities [4]. The lesions of the breasts detected by MRI in our proband were so extensive that obtaining histological samples from all the suspicious sites was impossible. An individual breast cancer risk assessment for a CS patient is complicated because of the rarity of the syndrome.

In some CS cases diagnostic difficulty is associated with dense breast tissue. This may lead to multiple biopsies with increased scarring during healing and further complicated physical examination and radiological evaluation. Moreover, the benign findings at core biopsies do not exclude that invasive carcinoma might arise within densely fibrotic, hamartomatous areas of the breast [12]. Psychological factors should be considered as well. The stress of constant surveillance and the threat of multiple biopsies may lead CS patients to choose bilateral risk-reducing mastectomy even if indications do not correspond to consensus guidelines.

In conclusion, as risk-reducing surgery is becoming a more acceptable option for high risk individuals, surgeons need to balance their decisions between individual risk assessment, potential psychological benefits and accepted guidelines [17].

If Cowden syndrome is confirmed by molecular examinations, risk-reducing bilateral mastectomy may be an effective preventive measure of breast cancer. This is to our best knowledge the first reported CS case with follow-up data after risk-reducing measures have been taken..

## Consent

A written consent was obtained from the patient before the publication of this case report. A copy of the written consent is available for review on request.

## Abbreviations

*PTEN*: Phosphatase and tensin homolog; CS: Cowden syndrome; NCCN: National Cancer Comprehensive Network; DNA: Deoxyribonucleic acid; MRI: Magnetic resonance imaging.

## Competing interests

The authors declare that they have no competing interests.

## Authors' contributions

IMG did the primary literature research and wrote the manuscript. IS and AA performed histopathological examinations. AI performed preoperative communication with the patient regarding risk-reducing surgery, the operations and postoperative treatment of the patient. SS and AG provided clinical consultations. EM and DB performed the *PTEN *mutation testing. AI and EM advised and assisted in drafting and writing the report. GT and JG coordinated the study. All the co-authors have read and approved the final version of the manuscript.
